# Ultra- and micro-structural changes of respiratory tracts in SARS-CoV-2 infected Syrian hamsters

**DOI:** 10.1186/s13567-021-00988-w

**Published:** 2021-09-16

**Authors:** Myeon-Sik Yang, Byung Kwan Oh, Daram Yang, Eun Young Oh, Yeonhwa Kim, Kyung Won Kang, Chae Woong Lim, Gou Young Koh, Sang-Myeong Lee, Bumseok Kim

**Affiliations:** 1 Laboratory of Veterinary Pathology, College of Veterinary Medicine, Jeonbuk National University, Iksan, 54596 South Korea; 2 Laboratory of Veterinary Virology, College of Veterinary Medicine, Chungbuk National University, Cheongju, 28644 South Korea; 3 Division of Biotechnology, College of Environmental and Bioresources, Jeonbuk National University, Iksan, 54596 South Korea; 4 Graduate School of Medical Science and Engineering, Korea Advanced Institute of Science and Technology (KAIST), Daejeon, 34141 South Korea

## Abstract

The severe acute respiratory syndrome coronavirus 2 (SARS‐CoV‐2) pandemic is causing a global crisis. It is still unresolved. Although many therapies and vaccines are being studied, they are still in their infancy. As this pandemic continues, rapid and accurate research for the development of therapies and vaccines is needed. Therefore, it is necessary to understand characteristics of diseases caused by SARS-CoV-2 through animal models. Syrian hamsters are known to be susceptible to SARS-CoV-2. They were intranasally inoculated with SARS-CoV-2. At 2, 4, 8, 12, and 16 days post-infection (dpi), these hamsters were euthanized, and tissues were collected for ultrastructural and microstructural examinations. Microscopic lesions were prominent in the upper and lower respiratory tracts from 2 and 4 dpi groups, respectively. The respiratory epithelium in the trachea, bronchiole, and alveolar showed pathological changes. Inflammatory cells including neutrophils, lymphocytes, macrophages, and eosinophils were infiltrated in/around tracheal lamina propria, pulmonary vessels, alveoli, and bronchiole. In pulmonary lesions, alveolar wall was thickened with infiltrated inflammatory cells, mainly neutrophils and macrophages. In the trachea, epithelial damages started from 2 dpi and recovered from 8 dpi, consistent with microscopic results, High levels of SARS-CoV-2 nucleoprotein were detected at 2 dpi and 4 dpi. In the lung, lesions were most severe at 8 dpi. Meanwhile, high levels of SARS-CoV-2 were detected at 4 dpi. Electron microscopic examinations revealed cellular changes in the trachea epithelium and alveolar epithelium such as vacuolation, sparse micro-organelle, and poor cellular margin. In the trachea epithelium, the number of cytoplasmic organelles was diminished, and small vesicles were prominent from 2 dpi. Some of these electron-lucent vesicles were filled with virion particles. From 8 dpi, the trachea epithelium started to recover. Because of shrunken nucleus and swollen cytoplasm, the N/C ratio of type 2 pneumocyte decreased at 8 and 12 dpi. From 8 dpi, lamellar bodies on type 2 pneumocyte cytoplasm were increasingly observed. Their number then decreased from 16 dpi. However, there was no significant change in type 1 pneumocyte. Viral vesicles were only observed in the cytoplasm of type 2 pneumocyte. In conclusion, ultra- and micro-structural changes presented in this study may provide useful information for SARS-CoV-2 studies in various fields.

## Introduction

With Severe Acute Respiratory Syndrome coronavirus (SARS-CoV) emerging in 2002, structure and surface proteins of SARS-CoV had been studied. The spike (S) protein of SARS-CoV is distinct from other coronaviruses. Many attempts have been made to find the host cell surface receptor for viral entry. Finally, a specific metallopeptidase, named angiotensin-converting enzyme 2 (ACE2), in the SARS-CoV-permissive Vero-E6 cell was identified as a cell entry receptor [1]. As a functional receptor for SARS-CoV infection, ACE2 is widely distributed in the body, primarily in pulmonary epithelial cells and enterocytes of the small intestine [2]. However, a recent study has indicated that the highest region of ACE2 expression is the nose and that ACE2 expression levels decreased in the lower respiratory tract [3], making an infectivity gradient for SARS-CoV. These findings provide possible routes of entry for understanding the pathogenesis of SARS-CoV. The ongoing pandemic, coronavirus disease 2019 (COVID-19), is caused by a novel SARS-CoV-2. Genetically, this newly emerging virus is closely related to SARS-CoV. SARS-CoV-2 also uses the SARS-CoV receptor ACE2 for host cell entry [4, 5]. Despite these similar characteristics, SARS-CoV-2 is less deadly but more transmissible than SARS-CoV. It had lasted over 1 year.

It is essential to find appropriate animal models to study the pathogenesis of infectious diseases. Since SARS-CoV-2 uses ACE2 for host cell entry, expression and recognition of this protein are important. For SARS-CoV-2 studies, the most used mammalian research model, mouse (*Mus musculus*), is insufficient because the sequence and structure of murine ACE2 differ from those of human. In addition, murine ACE2 does not effectively bind to the viral S protein of SARS-CoV-2 [6]. To solve this problem, a transgenic mouse model expressing human ACE2 (hACE2) has been developed [7, 8]. It has been shown that hACE2 transgenic mice show weight loss, histopathological lesions, and viral replication following SARS-CoV-2 infection [9]. However, hACE2 is also expressed in the brain. It is known that SARS-CoV-2 can spread to the brain via the olfactory bulb and cause neuronal death [10]. Syrian hamster (*Mesocricetus auratus*) is another rodent for laboratory studies. During the SARS-CoV pandemic, although the specific tissue expression of ACE2 has not been determined, susceptibility of hamster cells to SARS-CoV infection has been suggested [11] and histopathological evidence of the disease has been established in infected hamsters [12]. However, mild clinical symptoms and no mortality rate after viral infection in hamsters have been pointed out as the pros and cons, respectively, for using hamsters in SARS-CoV experiments [13]. In this regard, Syrian hamster has been used for studying SARS-CoV pathogenesis and antiviral drug testing. Spontaneously, when SARS-CoV-2 emerged and was shown to be genetically close and included in the same subgenus as SARS-CoV, Syrian hamster has been used for SARS-CoV-2 research. A recent study indicated that males are more susceptible than females for SARS-CoV-2 infection, with males showing higher histopathological scores, viral titers, and clinical symptoms than females [14]. Moreover, in an age-dependent study with Syrian hamsters, young hamsters (6-week-old) showed stronger immune cell influx and faster rapid recovery than old hamsters (32-to-34-week-old) after SARS-CoV-2 infection [15].

Since SARS-CoV-2 infects the respiratory tract, histopathological lesions are mainly observed in respiratory tracts of SARS-CoV-2 infected patients and animal models. In humans, type 2 pneumocyte hyperplasia, hyaline membrane, inflammatory cell infiltration, and fibrosis are the prominent pulmonary lesions [16]. In Syrian hamsters, alveolar edema, bronchopneumonia, interstitial pneumonia, and inflammatory cell infiltration are reported [17]. In the case of human pathology, the description and nomenclature of lesions are consistent in many SARS-CoV-2 cases and reports. However, in the case of Syrian hamster studies, the terminology and lesion description are not well defined. In addition, histopathological analysis of the upper respiratory tract has only been performed in a few studies, with most of them describing pulmonary lesions in Syrian hamsters [17, 18].

Ultrastructurally, when human airway epithelia are experimentally infected with SARS-CoV-2, numerous viral vesicles and double-membrane vesicles are observed 48 h post-infection (dpi) in transmission electron microscope (TEM) analysis [19]. Previous study has also shown ultrastructural changes of respiratory tracts in SARS-CoV-2 infected Syrian hamsters. When 6 × 10^4^ plaque-forming unit (PFU) of SARS-CoV-2 was intranasally inoculated, significant tracheal cilia loss was observed at 4 dpi in ultrastructural analysis using a scanning electron microscope (SEM) [20]. In other studies on immunosuppressed Syrian hamsters, cytoplasmic vacuoles, virus particles, and cilia loss have been observed in respiratory tracts at 13 dpi by TEM [21].

Although many studies have been performed on SARS-CoV-2 with Syrian hamsters, ultrastructure and microstructure changes have not been well established or categorized. Based on the previous and present studies, here we report ultra- and microstructural changes of respiratory tracts of SARS-CoV-2 infected Syrian hamsters at different timepoints post-infection.

## Materials and methods

### Cell culture and virus preparation

SARS-CoV-2 (NCCP43326) was obtained from Korea Disease Control and Prevention Agency and cultured in African green monkey kidney clone E6 (Vero-E6) cells. Vero-E6 cells were grown in Dulbecco’s modified Eagle’s medium supplemented with 10% fetal bovine serum, MEM non-essential amino acids, 2 mM l-glutamine, 100 Units/mL penicillin, and 0.1 mg/mL streptomycin (Hyclone, UT, USA) and cultured at 37 °C in a 5% CO_2_ incubator. Prepared virus stocks were concentrated by ultracentrifugation with 30% sucrose at 23 000 rpm for 5 h, using an ultracentrifuge (Optima XPN-100, Beckman Coulter, CA, USA). Pellets were suspended in phosphate-buffered saline pH 7.4. The viral titer was calculated by plaque assay. All experimental procedures were performed in a biosafety level III (BSL3) facility of the Korea Zoonosis Research Institute (KOZRI) at Jeonbuk National University.

### Animal infection and sample collection

All animal experiments including maintaining, infection, physical examination, and necropsy were performed in an animal BSL3 (ABSL3) facility of KOZRI at Jeonbuk National University. All researchers were approved and qualified for ABLS3 experiments by KOZRI and/or the Bioethics Information Center. All experimental procedures were reviewed and approved by the Animal Ethics Committee of Jeonbuk National University (Approval No. CBNU-2019-00260).

Eighteen male Syrian hamsters (*Mesocricetus auratus*) at 6-week-old were obtained from Central Lab Animal (Seoul, South Korea). Hamsters were maintained in ABSL3 conditions under optimal physical environments (24 ± 2 °C, 50 ± 5% humidity). There were six experimental groups according to dpi; 2 dpi, 4 dpi, 8 dpi, 12 dpi, 16 dpi, and control (Cont, no infection) (*n* = 3 per group). After three days of adaptation, 1 × 10^6^ PFU SARS-CoV-2 in a 100 μL volume was inoculated intranasally to each hamster under a light isoflurane anesthesia. Body weight and temperature were measured daily at the same time. On each dpi, three SARS-CoV-2 infected hamsters were euthanized. Necropsy was then performed for sample collection. Collected samples were fixed in 10% neutral buffered formalin.

### Viral titer

Plaque assays were performed to determine SARS-CoV-2 infectious titer. Briefly, Vero E6 cells were seeded in a 12-well plate at a density of 2 × 10^5^ cells/well and cultured overnight in a CO_2_ incubator. The supernatant of homogenized tissues was subjected to tenfold serial dilutions from 10^1^ to 10^6^. After incubating for 1 h, the supernatant was removed. Each well was washed with PBS. After adding 1.5 mL of overlay media, cells were cultured for 3 days in a CO_2_ incubator. After 3 days, cells were fixed with 1 mL of 4% formalin and stained with 0.4% of crystal violet in 70% methanol in PBS. Viral titer was calculated after counting the number of plaques.

### RNA extractions and quantitative RT-PCR (qRT-PCR)

To prepare the standard curve for SARS-CoV-2, cDNA was synthesized from viral RNA extracted from SARS-CoV-2 (NCCP43326) using a ReverTra Ace qPCR RT Kit (TOYOBO, Osaka, Japan) according to the manufacturer’s instructions. The cDNA used for preparing reverse transcription qPCR curve was amplificated by PCR using receptor-binding domain (RBD): forward primer, 5′-GCTCCATGGCCTAATATTACAAACTTGTGCC-3′; reverse primer, 5′-TGCTCTAGACTCAAGTGTCTGTGGATCAC-3′. PCR products were purified with a Wizard SV Gel and PCR Clean-Up System (Promega, WI, USA) and ligated with a pGEM-T-easy vector (Promega, WI, USA). Afterward, the ligated PCR product was directly used to transform *E. coli* DH5α competent cells. A single colony was then selected based on colony PCR and used to prepare plasmid DNA using DNA-spin plasmid DNA purification kit (Intron, Seoul, Korea). Extracted plasmid DNA was titrated by measuring the optical density with a spectrophotometer and a standard curve was generated from tenfold serial dilutions of the standard.

### Histopathology and immunohistochemistry

Collected and fixed samples were routinely processed and embedded in paraffin wax (Surgipath Paraplast, Leica Biosystems Inc., IL, USA). Formalin-Fixed Paraffine-Embedded (FFPE) tissue blocks were sectioned at 4 μm in thickness with a standard rotary microtome (HM-340E, Thermo Fisher Scientific Inc., MA, USA). Tissue sections were stained with hematoxylin and eosin (H&E) with a standard laboratory protocol.

Trachea and pulmonary abnormalities were scored following their representative microscopic lesions. Scoring criteria had a range of 0 to 3 by the severity or proportion: 0, non-to-rare or under 10%; 1, mild or 10 to 40%; 2, moderate or 40 to 70%; and 3, severe or over 70%. The trachea had three criteria: 1, inflammation of lamina propria; 2, cellular exudates in tracheal lumen; and 3, tracheal epithelium damage. The lung had six criteria: 1) inflammation of peribronchiolar region; 2) inflammation of perivascular region; 3) cellular exudates in bronchiolar lumen; 4) bronchiolar epithelium damage; 5) thickening of alveolar wall (interstitial pneumonia); and 6) hemorrhage. On scoring, care should be taken because each lesion was not evenly distributed in the tissue, showing various patterns. The final score of each criterion was shown as the sum, with a high score meaning more microscopic damage. These criteria were categorized and summarized in Table 1.

**Table 1:** **SARS-CoV-2 specific criteria of histopathologic examination scoring**

Organ	Type of lesions	Representative lesions
Trachea	Lamina Propria Submucosa	Inflammatory cell infiltration Edema
	Lumen	Potein-rich exudate Cellular exudate; inflammatory cell Cellular exudate; desquamated cell debris Hemorrhage
	Epithelium	Inflammatory cell infiltration Morphological change of epithelial cell Loss of epithelial cilia Loss of intercellular integrity Detached epithelium from basement membrane
Lung	Inflammation	
	Peribronchiolar	Inflammatory cell infiltration Parabronchiolar inflammation
	Perivascular	Inflammatory cell infiltration Hyperemia
	Bronchiole lumen	Protein rich exudate Cellular exudate Floating detached epithelial cell Infiltrate into alveolar space
	Bronchiole epithelium damage	Morphological change of epithelial cell Loss of epithelial cilia Detached epithelium from basement membrane
	Interstitial pneumonia	Thickened alveolar wall Inflammatory cell infiltration into alveolar wall Proportion of lesion
	Hemorrhage	Blood leakage from vessels into perivascular region alveolar space

Each parameter has a range of 0 to 3 by its severity or proportion.

0: normal or under 10%; 1: mild or 10 to 40%; 2: moderate or 40 to 70%; 3: severe or over 70%.

The sum of score may represent the severity of each organ.

For immunohistochemistry (IHC), a silane-coated slide was used for strong adhesiveness. To re-establish immunoactivity, antigen retrieval was conducted with citrate buffer pH 6.0 at 95 °C for 30 min and RT for 20 min. Sections were then incubated overnight at 4 °C with a SARS-CoV-2 nucleocapsid protein (NP) antibody (40143-V08B, Sino Biological, PA, USA) at a 1:500 dilution in antibody diluent (E09-300, GBI labs, WA, USA). To label SARS-CoV-2 NP, a horseradish peroxidase-conjugated anti-rabbit IgG antibody (MP-7500, Vector Laboratory, CA, USA) was used. The antibody was visualized with 3,3'-diaminobenzidine (DAB, SK-4105, Vector Laboratory, CA, USA) at the concentration recommended by the manufacturers. Tissues were counterstained with methyl green. All slides were examined (BX53, Olympus, Tokyo, Japan) and captured (DP80, Olympus, Tokyo, Japan) microscopically using a light microscope. All histopathologic examinations were conducted in a double-blind manner with trained pathologists.

To quantify IHC results, ten images were randomly captured from each tissue (*n* = 3 per group). Image analysis was performed using analysis TS Auto 5.1 (Olympus, Tokyo, Japan). The percentage of IHC positive area was analyzed in defined magnification field and area (400 magnification field, 0.144 mm^2^).

### Transmission electronic microscope for ultrastructure observation

Trachea and lung samples were fixed in a mixed fixative solution containing 2% paraformaldehyde and 2% glutaraldehyde dissolved in 0.05 M sodium cacodylate buffer (pH 7.2) at 4 °C. Tissues were post-fixed in 1% osmium tetroxide in 0.05 M sodium cacodylate buffer at 4 °C for 1.5 h. These post-fixed tissues were dehydrated through a graded series of ethanol and embedded in epoxy resin. After polymerization, blocks were trimmed and cut with an ultramicrotome (UC7, Leica Microsystems, Wetzlar, Germany). Then 1 μm semi-thin sections were stained with H&E for area selection under a light microscope. Thin (70 nm) sections were then stained with 2% uranyl acetate and Reynold’s lead citrate. Imaging analysis was performed at 100 kV using a TEM (H-7650, Hitachi, Tokyo, Japan). Digital images of specimens were taken using a Gatan Erlangshen 785 ES1000W CCD camera (Gatan Inc., CA, USA). All sample processing and analysis were conducted with the support of the Center for University-wide Research Facilities (CURF) at Jeonbuk National University.

### Statistical analysis

All data are expressed as mean ± standard deviation (SD). One-way analysis of variance (ANOVA) was used to test for statistical significance among experiment groups followed by Duncan’s multiple range test for multiple comparisons as a post hoc test. Significant differences among groups were marked by different letters on the graph. A *P* value < 0.05 was considered statistically significant. All statistical analyses were carried out using SAS statistical software (Version 9.4, SAS Institute Inc., Cary, NC, USA). All scoring and calculating data graphs were drawn with GraphPad Prism (Version 8.0.1, GraphPad Software, San Diego, CA, USA).

## Results

### Body weight loss of Syrian hamsters following SARS-CoV-2 infection

In our pilot study, histopathological examination was performed with various SARS-CoV-2 titers: 1 × 10^4^ PFU, 1 × 10^5^ PFU, and 1 × 10^6^ PFU. All infection groups showed higher histopathological scores for the nasal cavity and lung than the non-infection group. However, there were no significant differences in scores for nasal cavity or lung lesions among group with different viral titers. Among groups with different infection titers, the 1 × 10^6^ PFU titer group showed more significant differences in those histopathological scores compared to the non-infection group (unpublished data). Thus, the dose of 1 × 10^6^ PFU was used for SARS-CoV-2 infection in the present study.

**Figure 1:**
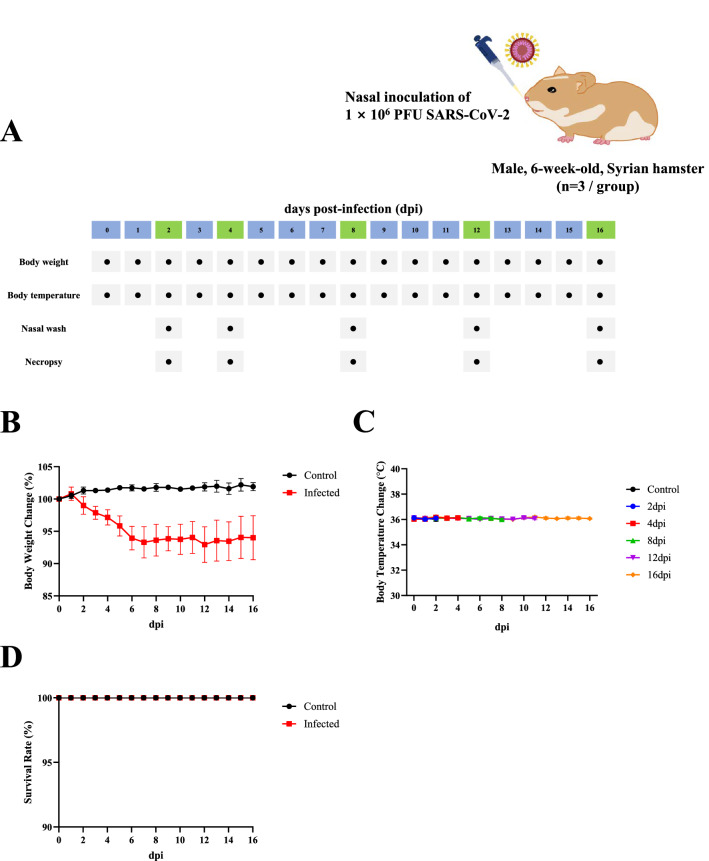
**Physical examination of SARS-CoV-2 infected Syrian hamsters (n = 3 per group).** A Schematic diagram of SARS-CoV-2 infection and animal operations. Six-week-old male Syrian hamsters were intranasally inoculated with 1 × 10^6^ PFU SARS-CoV-2. Body weight and temperature were measured daily. Nasal lavage and necropsy were performed at each time point. Changes of **(B)** body weight, **(C)** body temperature, and **(D)** survival rate are shown. All data were presented as means ± SD.

To observe ultra- and micro-structural changes of respiratory tracts, 6-week-old male Syrian hamsters were intranasally inoculated with 1 × 10^6^ PFU of SARS-CoV-2 (Figure 1A). Syrian hamsters infected with SARS-CoV-2 showed gradual weight loss until reaching a plateau at around 7 dpi. Such reduced body weight was maintained until 16 dpi (Figure 1B). Meanwhile, the body temperature did not change following virus infection until 16 dpi (Figure 1C). SARS-CoV-2 infection caused no mortality (Figure 1D).

### Viral clearance of SARS-CoV-2 during early time points after infection

**Figure 2:**
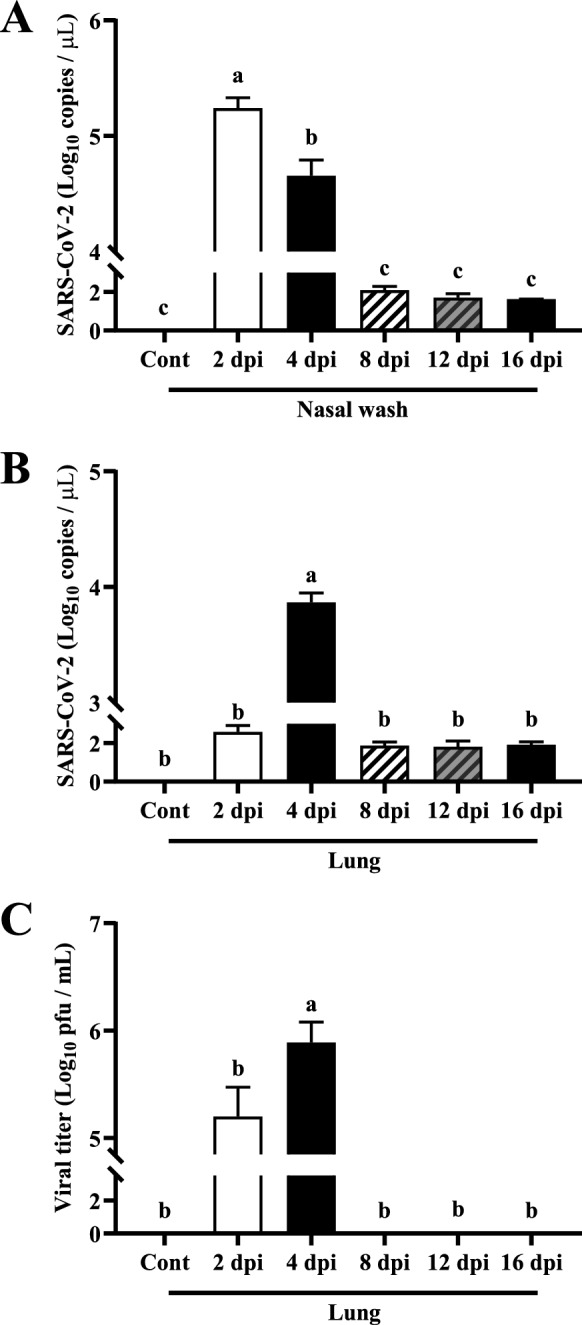
**Virus genome copy numbers in (A) nasal washes and (B) lung tissues determined by RT-qPCR.** The upper respiratory tract showed the highest levels of viral RNA at 2 dpi. Meanwhile, the lower respiratory tract showed the highest level of viral RNA at 4 dpi (*P* < 0.01). **C** Quantity of infectious viral particles in the tissues determined by plaque assay. The highest viral titer was observed at 4 dpi (*P* < 0.01). All data are presented as means ± SD (*n* = 3 per group). Viral RNA and titer are expressed in logarithmic scale using base 10 (Y axis).

SARS-CoV-2 RNA copy numbers in nasal washes and lung tissues were determined by RT-qPCR. High levels of viral RNA were detected in nasal washes (upper respiratory tract) and lung tissues (lower respiratory tract) at early time points after infection. The highest levels of viral RNA were detected at 2 dpi in nasal washes and 4 dpi in lung tissues, indicating that SARS-CoV-2 did not immediately infect the lower respiratory tract after intranasal inoculation (Figures 2A and B). Infectious viral particles in homogenized lung tissues were determined by plaque assay. Consistent with viral RNA results, high levels of viral titers were detected in lung tissues at 4 dpi (Figure 2C). However, viral RNA and viral titer were not detected from 8 dpi, indicating viral clearance from the respiratory tract.

### SARS-CoV-2 affects the trachea within 2 dpi, and the trachea recovers at around 8 dpi

**Figure 3:**
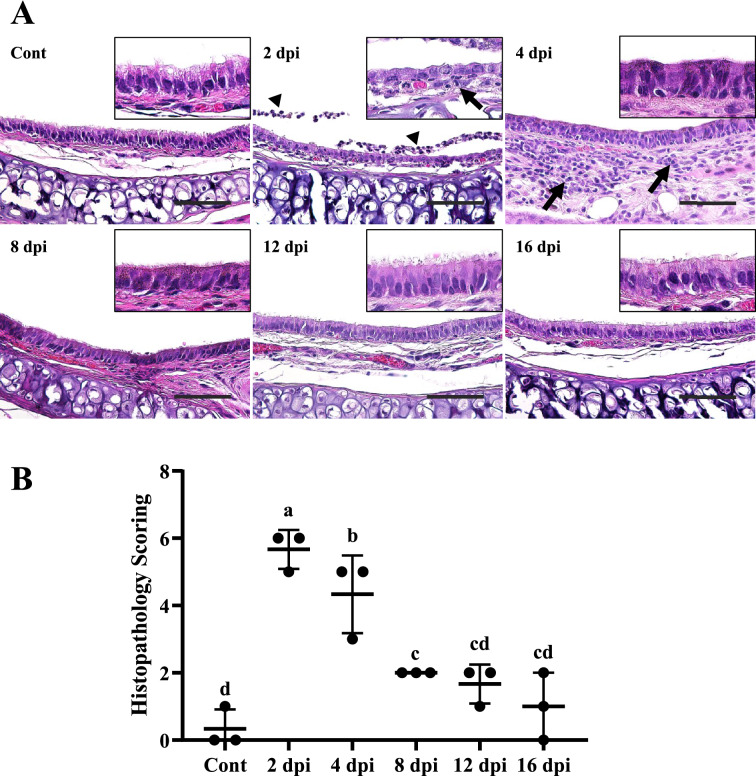
**Microscopic features of the trachea of male Syrian hamsters intranasally inoculated with 1 × 10**^**6**^** PFU of SARS-CoV-2 (n = 3 per group).A** Microscopic changes of trachea epithelium, lamina propria, submucosa, and lumen are shown. SARS-CoV-2 first affected the respiratory epithelium of the trachea at 2 dpi. The ciliated pseudostratified columnar epithelium of trachea was damaged and turned into squamous and/or cuboidal shaped cells. Cilia atop the respiratory epithelium lining were damaged and goblet cells were indistinct. These microscopic damages were recovered at 8 dpi. Inflammatory cells infiltrated into the lamina propria and submucosa layer from 2 dpi. This infiltration was the most severe at 4 dpi but was rarely observed at 8 dpi. Mononuclear inflammatory cells infiltrated into the lumen (black arrowheads) and lamina propria/submucosa (black arrows). H&E, Scale bar, 50 μm; Insert: higher magnification. **B** Histopathology scoring of the trachea. Data are presented as means ± SD. The microscopic lesion of trachea was the most severe in 2 dpi and 4 dpi groups compared to that in control and other infection groups (*P* < 0.01). Although 8 dpi, 12 dpi, and 16 dpi groups showed microscopic trachea damage, it was not significant compared to the control group.

At 2 dpi, the columnar shape of the tracheal epithelium turned into a squamous and/or cuboidal shape (Figure 3A). Damaged cilia were hardly distinctive. The epithelium was lined with a monolayer of cells with irregular size and shape. A mild cellular exudate was observed in the tracheal lumen. At 4 dpi, these anisokaryotic monolayer cells were transformed into anisokaryotic multilayer cells. The tracheal epithelium showed columnar changes compared to the 2 dpi group which had a relatively squamous and/or cuboidal shape. The epithelium was lined by a multilayer of cells with various size and shape. Cilia were not observed. Instead, microvilli were observed on the surface of the epithelium. Inflammatory exudates were not observed in the 4 dpi group. However, inflammatory cells such as neutrophil, macrophage, plasma cell, and lymphocyte were infiltrated into the lamina propria and the submucosa layer. From 8 dpi, the tracheal epithelium recovered. Cilia were retrieved atop the epithelium. The epithelium showed columnar shape with a monolayer. However, goblet cells were not observed distinctly. A few inflammatory cells still infiltrated into the lamina propria and the submucosa layer. At 12 and 16 dpi, the tracheal epithelium was completely recovered with well-defined goblet cells. These microscopic changes of the trachea were evaluated and quantified according to a microscopic scoring system (Table 1). Tracheal damage was observed at all time points after infection (Figure 3B). Groups of 2 dpi and 4 dpi showed higher scores than other groups (control, 8 dpi, 12 dpi, and 16 dpi) (*P* < 0.01), although there were no significant differences among 8, 12, and 16 dpi groups compared to the control group. These results indicated that infection of SARS-CoV-2 affected the trachea at the early stage after the infection.

**Figure 4:**
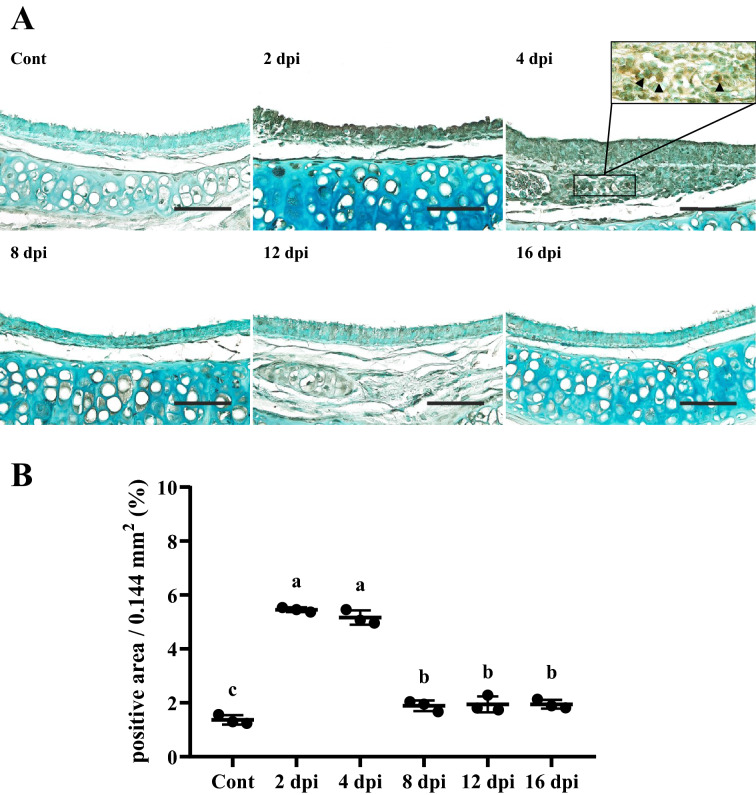
**IHC results of the trachea.A** Tracheal epithelium was strongly positive against SARS-CoV-2 nucleocapsid protein (NP) at 2 and 4 dpi. IHC positive mononuclear inflammatory cells were observed in the lamina propria (black arrowheads). Counterstain with methyl green. Scale bars, 50 μm; Insert: higher magnification (*n* = 3 per group). **B** Quantification of IHC positive area in the trachea against SARS-CoV-2 NP. Data are presented as means ± SD. Both 2 dpi and 4 dpi groups showed strong positive areas compared to control and other infection groups (*P* < 0.01). Positive areas in 8, 12, and 16 dpi groups were strongly detected compared to the control group (*P* < 0.01) (*n* = 3 per group).

On IHC examination, 2 dpi and 4 dpi groups showed more positive reactions against SARS-CoV-2 NP in the epithelium compared to control and other infection groups (*P* < 0.01). At 4 dpi, infiltrated mononuclear inflammatory cells in the lamina propria and submucosa were positively stained for SARS-CoV-2 NP. This might be because macrophages phagocytosed viral pathogens. Meanwhile, at 8, 12, and 16 dpi, positive area was slightly more detected compared to the control group (*P* < 0.01) (Figures 4A and B).

**Figure 5:**
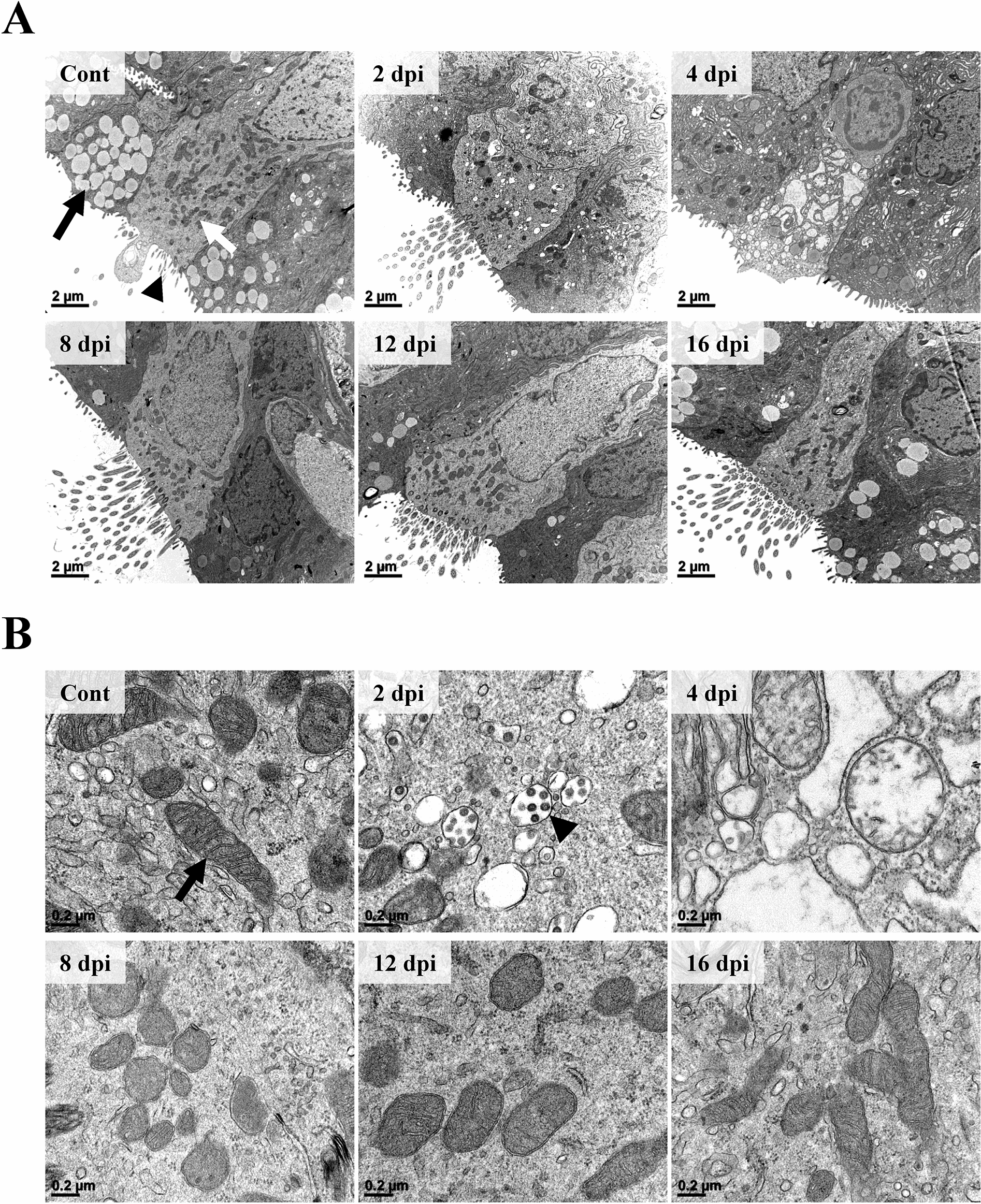
**Ultrastructural features of the trachea epithelium following infection (n = 3 per group).A** The ciliated pseudostratified columnar epithelium showed ultrastructural changes from 2 dpi. Gradual changes of secretory vacuoles (black arrow), cytoplasmic organelles (white arrow), and cilia (black arrowhead) were prominent following infection. H-7650. TEM. 100 kV. Scale bars, 2 μm. **B** The density and morphology of cytoplasmic organelles were changed from 2 dpi. The outer membrane and the cristae membrane of mitochondria (black arrow) showed morphological changes. At 2 and 4 dpi, various sizes of electron-lucent spaces were observed. Note that virion-containing vesicles were observed in the cytoplasm at 2 dpi (black arrowhead). H-7650. TEM. 100 kV. Scale bars, 0.2 μm.

In ultrastructural inspection through electron microscopy (EM) observation, damage and recovery of ciliated pseudostratified columnar epithelium of trachea were clearly observed. In the control group, the epithelium was elongated in a columnar shape (Figure 5A). The endoplasmic reticulum (ER) and cytoplasmic organelles were dense and clear. The electron-dense mitochondria had clear outer membrane. The cristae membrane was well-margined and distinct (Figure 5B). Besides the epithelium, electron-lucent secretory vacuoles of matured goblet cells were observed. Cilia and microvilli were observed on the surface of the epithelium. At 2 dpi, the epithelium was shortened compared to that in the control group and the nucleus was shrunk with irregular margins. Secretory vacuoles in goblet cells located adjacent to ciliated pseudostratified columnar epithelium were not observed in the 2 dpi group. ER and cytoplasm were indistinct and various sizes of vesicles were observed. The density of cytoplasmic organelles including mitochondria was diminished and mitochondria had a poor membrane margin. Some vesicles were filled with SARS-CoV-2 virions. Virions had size of 50 to 80 nm with a rough spherical morphology. The virion surface was projected with spike shape structures that were embedded in an envelope and visible as small electron-dense dots. At 4 dpi, cytoplasm and ER were replaced by various sizes of vacuoles and vesicles, making a labyrinth pattern. These cells were extruded at the surface of the epithelial lining. Cilia, secretory vacuoles, and cytoplasmic organelles including mitochondria were not observed. At 8 dpi, epithelium recovery was observed. Cilia and mitochondria were observed. However, cytoplasmic vesicles were not observed. Some secretory vacuoles started to be observed in adjacent goblet cells. However, ER was unclear and mitochondrial membrane was still ambiguous. At 12 and 16 dpi, ER and mitochondrial membrane became more obvious, and many ribosomes and mitochondria were observed.

Based on these ultra- and microstructural examinations of the trachea, it was confirmed that SARS-CoV-2 affects and damages the trachea at the early stage of the infection. Recovery and viral clearance of the trachea were observed at 8 dpi.

### The most severe lesions in the lung are observed at around 8 dpi

**Figure 6:**
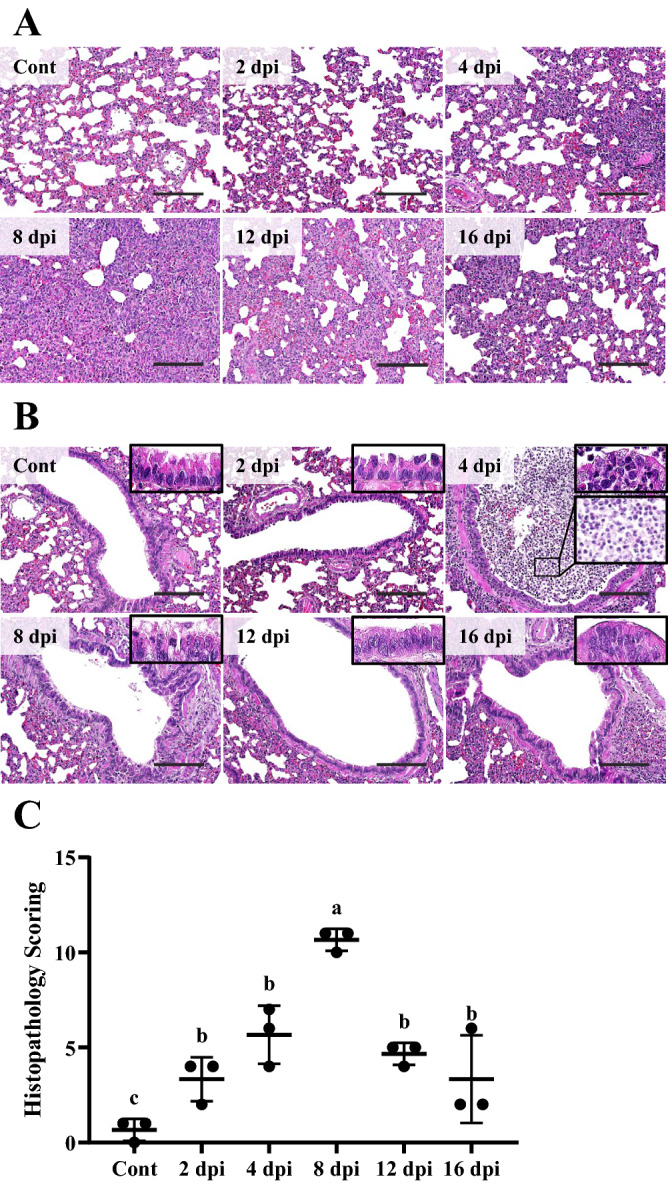
**Microscopic features of the lung from male Syrian hamsters intranasally inoculated with 1 × 10**^**6**^** PFU of SARS-CoV-2 (n = 3 per group).A** Consolidations of lung parenchyma at various degrees were shown after SARS-CoV-2 infection. Prominent alveolar wall thickening was observed from 4 dpi with perivascular inflammatory cell infiltration. Consolidation was the most severe at 8 dpi. These severe proliferative lesions became relatively moderate at 12 and 16 dpi. H&E. Scale bars, 100 μm. **B** Microscopic changes of the bronchiolar epithelium, lumen, submucosa, and peribronchiolar region. Exudates with inflammatory cells and cell debris were observed in the bronchiolar lumen at 4 dpi. These damages of the epithelium such as cilia loss, vacuolation, and proliferation were various following the infection. H&E. Scale bars, 100 μm; Insert: higher magnification. **C** Histopathology scoring of the lung. Data are presented as means ± SD. Microscopic lesions of lung showed significant differences in 4 and 8 dpi groups than the control group (*P* < 0.01). The most severe lesion was observed at 8 dpi among infection groups (*P* < 0.01). No significant differences in lesion were observed among 2, 4, 12, and 16 dpi groups.

At 2 dpi, the lung parenchyma was intact. Alveolar wall thickness was not prominent. Inflammatory cell infiltration was not observed either (Figure 6A). However, in the bronchioles, the epithelium showed morphological changes (Figure 6B). The cytoplasm was eosinophilic. The intercellular gap junction was widened. Cilia were not observed compared to the control group. Inflammatory cell infiltration in the peribronchiolar region or the bronchiolar lumen was not observed. At 4 dpi, moderate alveolar wall thickness (interstitial pneumonia) was observed. With thickened alveolar walls, inflammatory cells infiltrated into the perivascular region and the alveolar wall. Bronchiole lumen was filled with dense inflammatory exudates. Inflammatory cells also infiltrated into the lamina propria, submucosa, and peribronchiolar regions. Among these infiltrated inflammatory cells, neutrophils and macrophages were the most populated. Interestingly, among various types of inflammatory cells, abundant eosinophils were recruited into the bronchiolar lumen. Some of bronchiolar epithelium showed squamous and/or cuboidal shape and mild proliferation. A vacuole-like structure was observed in some epithelial cells. Cilia were hard to recognize and cell margins were poor. In the 8 dpi group, interstitial pneumonia was severe. Because of proliferative changes of type 2 pneumocytes and infiltrated inflammatory cells, the lung parenchyma lost its proper mesh-like alveoli structure. In bronchioles, mild cellular exudates were observed in the lumen and the surface of the epithelial lining. Most epithelia were columnar. However, some squamous and/or cuboidal cells were observed. Some epithelia showed a multilayered lining with mild proliferation. Cell margins were still unclear. Inflammatory cells mildly infiltrated into the peribronchiolar region. In 12 and 16 dpi groups, interstitial pneumonia was observed. However, lesions were moderate and less severe compared to those in the 8 dpi group. Bronchiolar epithelium showed clear cell margins and a columnar shape. Inflammatory cells did not infiltrate into the lumen or the peribronchiolar region. These microscopic changes of the lung were evaluated and quantified according to a microscopic scoring system. The 8 dpi group showed the most severe pulmonary damages among all groups (*P* < 0.01) (Figure 6C). Meanwhile, the 4 dpi group showed more severe pulmonary damage than the control group (*P* < 0.01). However, it showed no significant differences in pulmonary damage compared to 2, 12, and 16 dpi groups. These results indicated that SARS-CoV-2 affected the lung with prominent inflammatory and/or proliferative reactions and that these pulmonary damages were the most severe at around 8 dpi.

**Figure 7:**
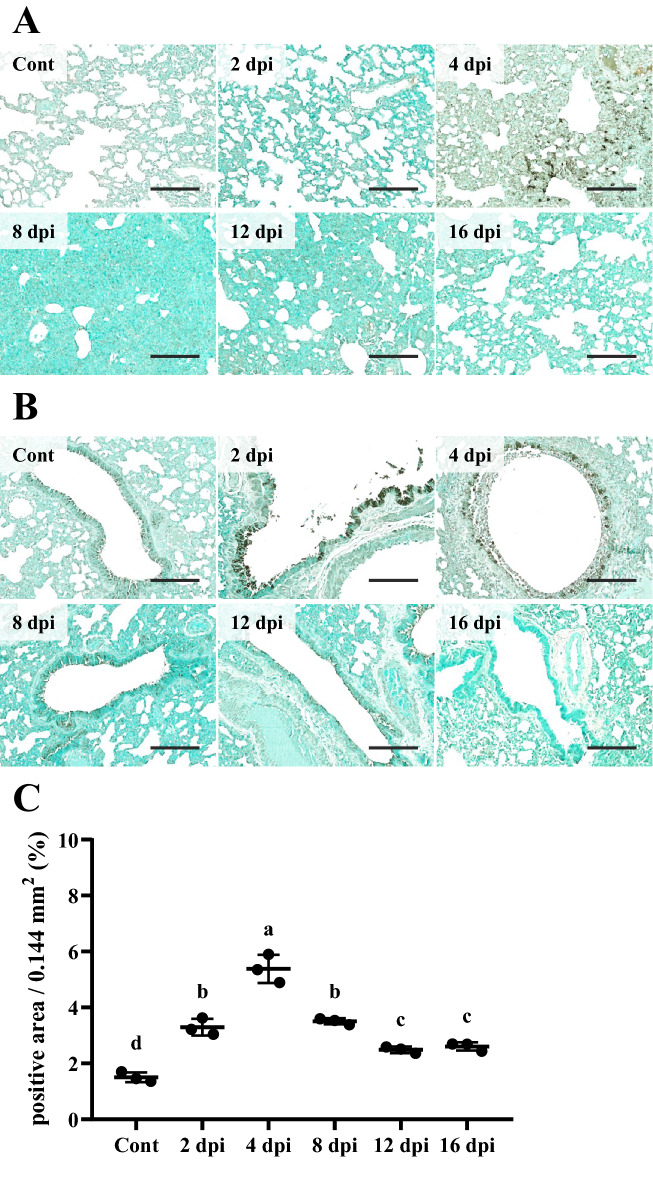
**IHC results of the lung.A** Lung parenchyma was strongly positive against SARS-CoV-2 NP in IHC at 4 dpi. Counterstain with methyl green. Scale bars, 100 μm (*n* = 3 per group). **B** Bronchiolar epithelium showed strong positive against SARS-CoV-2 NP in IHC on 2 and 4 dpi. Counterstain with methyl green. Scale bars represent 100 μm. **C** Quantification of IHC positive area in lung against SARS-CoV-2 NP. Data are presented as means with SD. All infection groups showed higher positive areas than the control group (*P* < 0.01). The 4 dpi group showed more strong positive area compared to the control and other infection groups (*P* < 0.01). IHC positive areas in 12 and 16 dpi groups were lower compared to those in other infection groups (*P* < 0.01) (*n* = 3 per group).

On IHC examination, strong IHC positive reaction against SARS-CoV-2 NP started to be observed on bronchiolar epithelial cells at 2 dpi without positive reaction on the alveolar epithelium (Figures 7A and B). At 4 dpi, bronchiolar epithelial cells and luminal inflammatory cells showed IHC positive reactions. These positive areas started to be observed on alveolar epithelial cells. Meanwhile, at 8, 12, and 16 dpi, a small portion of the positive area was detected. When IHC results were quantified, all infection groups showed higher proportion of the IHC positive area compared to the control group (*P* < 0.01) (Figure 7C). Among infection groups, the 4 dpi group showed the highest percentage of IHC positive area against SARS-CoV-2 NP (*P* < 0.05). Meanwhile, 12 and 16 dpi groups showed lower percentages of positive area compared to 2, 4, and 8 dpi groups (*P* < 0.01).

**Figure 8:**
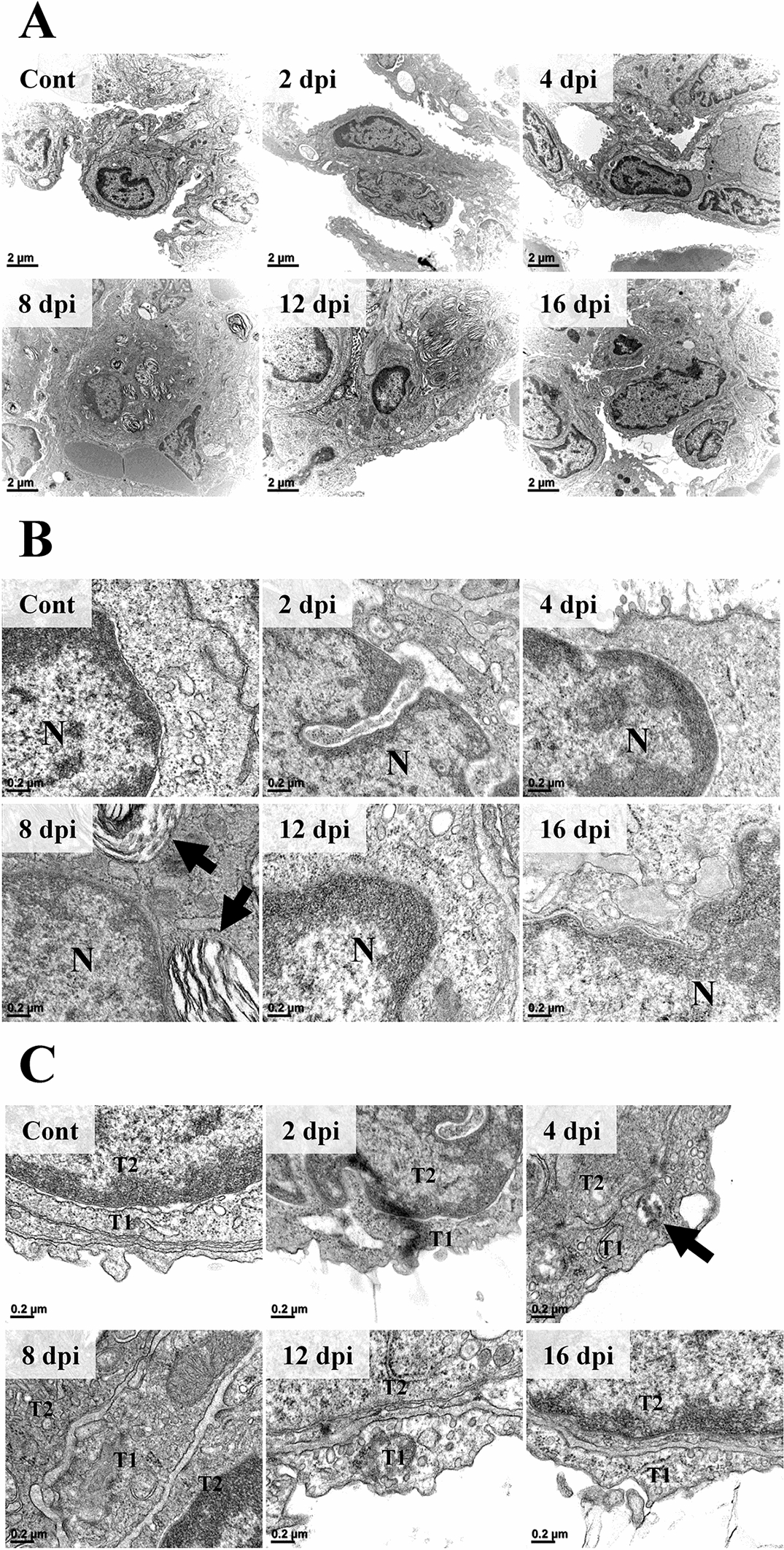
**Ultrastructural features of the alveolar epithelium following infection (*n*= 3 per group). A** The ultrastructure of type 2 pneumocytes. At 8 dpi, the N/C ratio was decreased compared to that in the control, 2 dpi, or 4 dpi. Lamella bodies in the cytoplasm at 8 dpi were remarkable. These lamella bodies were decreased at 16 dpi compared to that at 8 and 12 dpi. H-7650. TEM. 100 kV. Scale bars, 2 μm. **B** The ultrastructure of type 2 pneumocyte nucleus and adjacent cytoplasm. The nuclear membrane was not clear. It then recovered following the infection period. Lamella bodies observed at 8 dpi were remarkable (black arrow). H-7650. TEM. 100 kV. Scale bars, 0.2 μm. **C** The ultrastructure of type 1 pneumocyte. The type 1 pneumocyte did not show prominent changes following SARS-CoV-2 infection regardless of infection period. Note that virion-containing vesicles were observed in the cytoplasm at 4 dpi (black arrow). T1, type 1 pneumocyte; T2, type 2 pneumocytes. H-7650. TEM. 100 kV. Scale bars, 0.2 μm.

In ultrastructural observation under EM, type 2 pneumocytes of control and 2 dpi groups showed an intact cellular structure (Figure 8A). The nuclear membrane was distinctly separated from the cytoplasm (Figure 8B). The electron-dense nucleolus and chromatin inside the nucleus were well-defined and cytoplasmic organelles were intact. The alveolar space which was visible as an electron-lucent area, encircled the alveolar epithelium. At 4 dpi, the cellular ultrastructure was still intact. However, the nuclear membrane was not sharply demarcated. The number of ribosomes visible as small electron-dense dots was diminished compared to control and 2 dpi groups. As pneumocytes proliferated and projected into the alveolar space, the area of the alveolar space was diminished. At 8 dpi, type 2 pneumocytes were swollen and the nuclear to cytoplasmic ratio (N/C ratio) was decreased compared to that in the control, 2 dpi, or the 4 dpi group. The nuclear membrane was still indistinct. However, the decrease of ribosome in the 4 dpi group recovered. Interestingly, lamellar bodies and secretory organelles were observed in the cytoplasm, and eosinophil infiltration into the alveolar epithelial lining was observed at 8 dpi. At 12 dpi, the nuclear membrane was unclear and lamellar bodies were still observed. Ultrastructural cellular changes including nuclear membrane, N/C ratio, and cytoplasmic organelles recovered to those of the control group at 16 dpi. However, a few lamellar bodies were still observed in the cytoplasm. Meanwhile, ultrastructural changes of type 1 pneumocytes were not prominent after SARS-CoV-2 infection regardless of the infection period (Figure 8C). However, at 4 dpi, SARS-CoV-2 virion vesicle was observed in the cytoplasm of type 1 pneumocyte.

**Figure 9:**
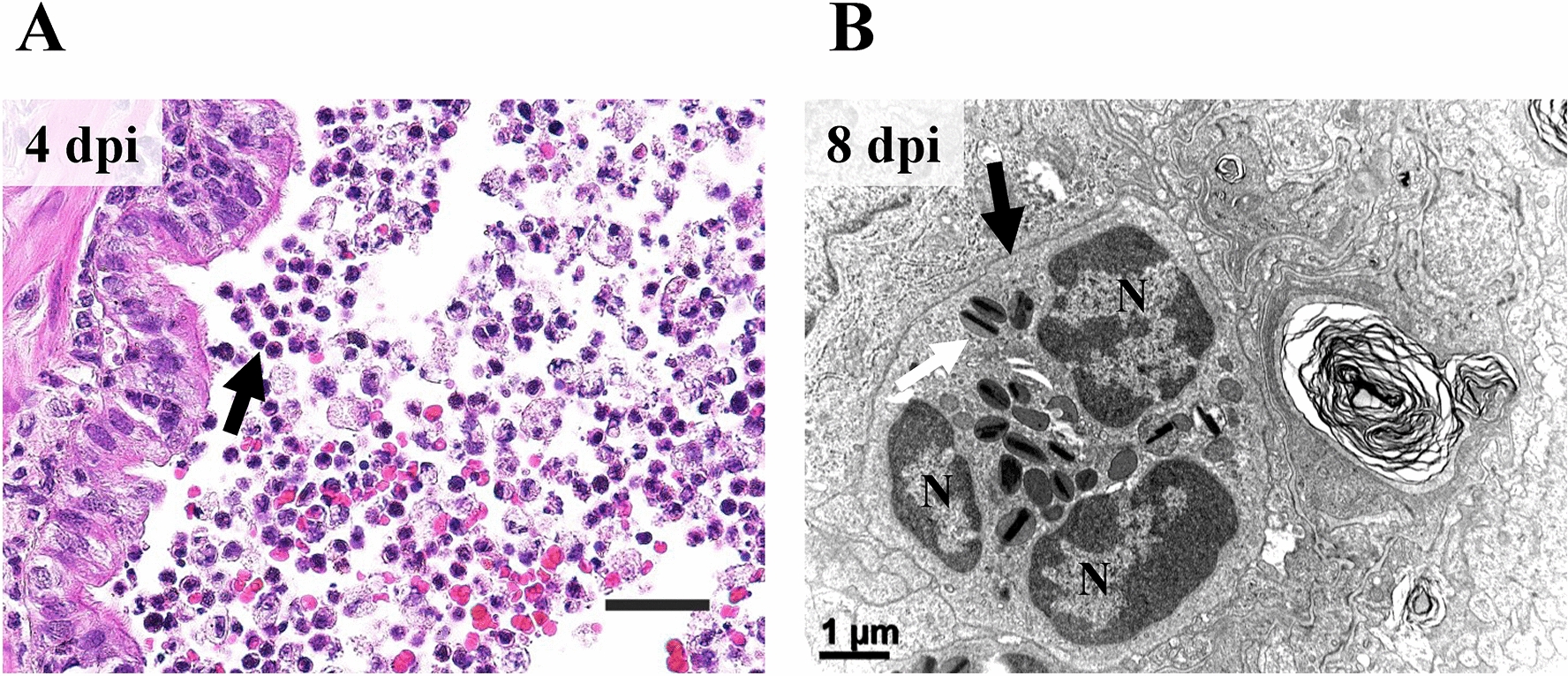
**Eosinophil recruitment and infiltration.A** Recruitment of eosinophils on the surface of bronchiolar epithelial lining (black arrow). Bronchiolar lumen at 4 dpi. H&E. Scale bar, 20 μm. **B** The ultrastructure of infiltrated eosinophil (black arrow) into the alveolar epithelium lining at 8 dpi. Segmented nucleus and electron-dense crystalloid body, called specific granules (white arrow), were observed. N, nucleus. H-7650. TEM. 100 kV. Scale bar, 1 μm.

Of infiltrated inflammatory cells, varying degrees of eosinophils were notably observed. The recruitment of eosinophils was prominent in the bronchiolar lumen at 4 dpi (Figure 9A). At 8 dpi, infiltrated eosinophil in the alveolar wall was confirmed under ultramicroscopic examination (Figure 9B).

These ultra- and microstructural features of the lung confirmed that SARS-CoV-2 infection damages the bronchiolar epithelium first, and the alveolar epithelium later. Bronchiolar epithelium was affected at around 2 dpi and inflammatory cells infiltrated the perivascular and bronchiole region at around 4 dpi. Interstitial pneumonia lesions were prominent at around 8 dpi. These pathological changes of the alveolar epithelium were more prominent in type 2 pneumocytes than in type 1 pneumocytes.

## Discussion

In 2020, many studies have been conducted to evaluate SARS-CoV-2 related pathogenicity using Syrian hamsters. Aims of these studies were to overcome the current COVID-19 crisis quickly and safely. SARS-CoV-2 experiments have been performed using Syrian hamsters of diverse age and sex and various virus titers and infection periods. Some of these experiments include histopathological examinations. To analyze the effect of SARS-CoV-2 on hamster respiratory tracts, histopathological evaluations of the upper and/or lower respiratory tract were performed in this study. Such histopathological examination is intuitive, relatively fast, and accurate. However, depending on the level of proficiency of the examiner, results may not be objective. Results may also vary depending on evaluation parameters that the examiner considers to be important. For example, a person who places a high value on changes in cellular level and/or functional structure of an organ might have different results. Before this experiment, our team had performed several experiments related to SARS-CoV-2 using Syrian hamsters (unpublished data). In those experiments, we observed several common and representative microscopic lesions in respiratory tracts. To standardize and quantify histopathological examinations, a microscopic scoring system was needed for SARS-CoV-2 study with Syrian hamsters. To obtain the scoring system, eight recently published papers having histopathological findings for SARS-CoV-2 infected Syrian hamster were analyzed [15, 18, 22–27]. Unfortunately, histopathological parameters and descriptions in these eight papers were inconsistent. Examination sites varied across the respiratory system. There were also differences in the way of lesion description. Their scoring systems were not consistent either. Some were too detailed and specific, while others were too concise or rough for describing these lesions. We thought that a new scoring system should be given. Thus, we summarized parameters used in these published papers for reference (Table 2). We considered to include lesions commonly observed in reported studies and our previous experiments, as much as possible. Thus, we integrated them into a concise and easy evaluating way. In this regard, we suggested an objective scoring system for SARS-CoV-2 study using Syrian hamsters (Table 1).

**Table 2:** **Reported respiratory lesions on SARS-CoV-2 infected Syrian hamsters**

Histopathological parameter		Day post-infection						
		2	3	4	5	6	7	10
**Trachea**								
Epithelium	Shape change	[25]	[18]		[18]			
	Cilia loss	[25]						
	Proliferation						[25]	
	Necrosis		[18]		[18]			
	Inflammatory cell	[25]						
	Recover						[25]	
	Desquamation	[25]		[25]				
Lumen	Exudate							
	Hemorrhage							
	Inflammatory cell							
	Cell debris	[25]						
Lamina propria / Submucosa	Inflammatory cell	[25]		[25]				
	Edema							
**Lung**								
Inflammation		[23, 25]		[25]	[23]			
Bronchointerstitial pneumonia		[15]			[15]		[15]	
Bronchiole								
Epithelium	Shape change			[25]				
	Cilia loss							
	Proliferation				[15, 24, 27]		[18, 25]	
	Necrosis	[22]	[18, 27]		[18]			
	Inflammatory cell	[22]	[18, 27]	[25]	[18, 24, 27]			
Lumen	Exudate			[25]				
	Hemorrhage							
	Inflammatory cell	[22]	[27]		[24, 27]			
	Cell debris	[22, 25]		[25]				
Peribronchiole	Inflammatory cell			[25]	[24]			
**Alveoli**								
Epithelium	Proliferation	[22]	[18, 27]	[25]	[18, 24, 27]		[18, 23, 25]	
	Necrosis			[25]				
	Inflammatory cell		[18, 26]	[26]	[18, 24]	[26]		[26]
Space	Edema	[25]	[18, 26]	[25]	[18]	[26]		[26]
	Inflammatory cell	[25]	[26]	[25]	[18, 24]	[26]		[26]
	Hemorrhage		[26]	[25]		[26]		[26]
**Pulmonary Vessel**								
Perivascular cuffing					[18, 24]			[18]
Congestion/Hyperemia					[24]			
Hemorrhage								
damage		[22]			[24]			

Numbers in parentheses refer to reference numbers.

In human pathology, many COVID-19 confirmed cases from postmortem samples and biopsies were analyzed. In one review paper [28], histopathological lesions observed in 226 autopsies and 9 biopsy tissues were summarized. These histopathologic features have three major patterns in the lung: diffuse alveolar damage, lymphocyte infiltration, and microthrombi/thrombi. Of them, diffuse alveolar damage has three phases according to lesions: exudative, proliferative, and fibrotic phase. Although there are differences in degree and description, such diffuse alveolar damage and lymphocyte infiltration are also observed in experimentally infected hamsters. However, microthrombi and/or thrombi have not been reported [17]. Endothelial damage of pulmonary vessels in SARS-CoV-2 infected hamsters has been reported. However, thrombi have not been reported in hamsters [22, 24]. In this regard, we hypothesized the following reasons to explain why pulmonary thrombi were not well observed in hamsters. First, hamsters can recover rapidly from a SARS-CoV-2 infection (in about two weeks) [7]. In this regard, it is difficult to observe endothelial necrosis and fibrotic changes in blood vessels. Without these major elements of thrombosis, it is not an easy environment for thrombus formation. Also, in hamsters, severe vascular damage, angiogenesis, and recanalization were not reported. Second, hamsters are kept in a specific-pathogens-free area during the experiment. Humans are always exposed to pathogens until they are confirmed and isolated for SARS-CoV-2 infection. In this unspecified environment, lesions and patterns of infection are thought to be different from those in a controlled environment. Third, the effect of SARS-CoV-2 on vascular endothelial cells in a hamster is currently not obvious. In humans, direct vascular endothelial cell infection and resulting vasculitis have been reported [29]. Although ACE2 is also widely expressed on epithelial cells as on pneumocytes, it is currently ambiguous why the virus replicates better in respiratory tracts than in vascular endothelial cells [30]. To prove this, other SARS-CoV-2 receptors on the surface of human cells have been suggested, such as transmembrane serine protease 2, sialic acid, and extracellular matrix metalloproteinase inducer [31]. Otherwise, it has been reported that massive recruitment of inflammatory cells and immune mediators can accelerate blood vessel damage and dysfunction [29, 32]. In this regard, further studies are needed since hamsters might have a different mechanism of vascular endothelial cell infection of SARS-CoV-2 virus in the respiratory tract compared to humans. In conclusion, lesions observed throughout the respiratory system including the lung parenchyma in Syrian hamsters are similar to those in humans except that vascular lesions are relatively less. Therefore, the value of hamsters as an animal model in SARS-CoV-2 studies is still high. Further studies are needed to demonstrate mechanisms of weak vascular lesions.

Lamellar bodies, dense multi-layered cytoplasmic organelles in type 2 pneumocytes, have secretory function into the alveolar space. The component of the lamellar body is phospholipid which is the main material of pulmonary surfactant [3]. Pulmonary surfactant can lower the surface tension of the alveoli. It is also involved in immune responses. Among four types of surfactant-associated proteins (SP), hydrophilic SP-A and SP-D play a major role in host defensive innate immune response [33]. These pulmonary collections can bind to viruses and bacteria, thus inhibit their growth, replication, and entry [34]. Interestingly, SP-D is known to be able to recognize and bind to SARS-CoV, like other viruses such as influenza A virus, herpes simplex virus type 1, human immunodeficiency virus, and respiratory syncytial virus [35]. Serum SP-D levels are also higher in patients with COVID-19. They are decreased after therapeutic treatment [36]. In the present study, lamellar bodies were not observed on type 2 pneumocytes of the control group in the examined ultramicroscopic field. Lamellar bodies were not observed in 2 or 4 dpi groups either. These results indicate that lamellar bodies, which are normally present in type 2 pneumocytes to maintain surface tension of the lung, are not activated in force without physiological and/or pathological pulmonary damages. Furthermore, it was confirmed that SARS-CoV-2 did not cause enough lung damage to cause the activation of lamellar bodies at 2 or 4 dpi. Meanwhile, cytoplasmic lamellar bodies in type 2 pneumocytes were increasingly observed in the 8 dpi group in TEM examination compared to non-infection, 2 and 4 dpi groups. In the 16 dpi group, lamellar bodies were decreased compared to those in 8 and 12 dpi groups. In the aspect of lowering superficial tension of pulmonary surfactants, it made sense that lamella bodies were activated to make gas exchange more efficient in lungs following severe interstitial pneumonia at 8 dpi. Indeed, there have been trials of administering exogenous pulmonary surfactant to those with acute respiratory distress syndrome including SARS-CoV-2 infection [37, 38]. However, studies on antiviral and anti-inflammatory effects of lung surfactants on SARS-CoV-2 infection have not been reported yet. In addition, in the present study, lamella body was not observed at 4 dpi where the virus titer was the highest in the lung. To investigate whether lamella body is relevant to SARS-CoV-2 pathophysiology or whether it takes time for the activation of lamella body, further study is needed.

Eosinophils are granulocytes that belong to the innate branch of the immune system. Eosinophils have a similar phenotype and function as other granulocytes. However, they are responsible for their own domain by having unique granules called cationic granule proteins. Especially during viral infections, eosinophils may have a protective role against RNA viruses because granule proteins of eosinophils include abundant ribonucleases [39]. Indeed, a previous study has indicated that eosinophils have an antiviral role against some respiratory viruses such as respiratory syncytial virus, human rhinovirus, human parainfluenza virus, and influenza virus [40]. However, antiviral effects of eosinophil against coronavirus have not been shown. One paper has reported a decrease in peripheral blood eosinophils (eosinopenia) in COVID-19 confirmed patients. However, other papers suggested that eosinopenia might not be associated with the progression of COVID-19 [41, 42]. In this regard, the observation of eosinophils in alveolar epithelium lining and bronchiolar lumen in the present study should be interpreted carefully. Because eosinophils are relatively rare in the normal lung [43], our observation is thought to be a notable finding. Although the relationship between eosinophils and SARS-CoV-2 is currently not obvious, recruitment and infiltration of eosinophils are evident in the present study. Therefore, the observation of eosinophils on histopathological examination might be related to SARS-CoV-2 infection in Syrian hamsters. In a further study, experiments to count the number of eosinophils in bronchoalveolar lavage fluid and/or quantify the eosinophil through IHC need to be performed.
